# Post-cardiac surgery fungal mediastinitis: clinical features, pathogens and outcome

**DOI:** 10.1186/s13054-022-04277-6

**Published:** 2023-01-06

**Authors:** Geoffroy Hariri, Mathieu Genoud, Vincent Bruckert, Samuel Chosidow, Emmanuel Guérot, Antoine Kimmoun, Nicolas Nesseler, Emmanuel Besnier, Fabrice Daviaud, David Lagier, Julien Imbault, David Grimaldi, Adrien Bouglé, Nicolas Mongardon

**Affiliations:** 1 Département d’anesthésie et réanimation, Institut de Cardiologie, GRC 29, Assistance Publique-Hôpitaux de Paris (AP-HP), DMU DREAM, Hôpital La Pitié-Salpêtrière, Sorbonne Université, 75013 Paris, France; 2 Institut Pierre Louis d’épidémiologie et de santé publique, Inserm U1136, Sorbonne Université, 75013 Paris, France; 3 Service des urgences, Département de médecine aiguë, Hôpitaux Universitaires de Genève, 1205 Geneva, Switzerland; 4 Service d’anesthésie-réanimation, CHU de Nice, 06000 Nice, France; 5 Service d’anesthésie-réanimation chirurgicale, DMU CARE, DHU A-TVB, Assistance Publique-Hôpitaux de Paris (AP-HP), Hôpitaux Universitaires Henri Mondor, 94000 Créteil, France; 6 Médecine intensive-réanimation, Hôpital Européen Georges Pompidou, Assistance Publique-Hôpitaux de Paris (AP-HP), 75015 Paris, France; 7 CHRU de Nancy, Médecine intensive-réanimation Brabois, Inserm U1116, Université de Lorraine, 54000 Nancy, France; 8 Service d’anesthésie-réanimation, CHU de Rennes, 35000 Rennes, France; 9 CHU de Rennes, Inra, Inserm, Institut NUMECAN – UMR_A 1341, UMR_S 1241, CIC 1414 (Centre d’Investigation Clinique de Rennes), Univ Rennes, 35000 Rennes, France; 10 Département d’anesthésie-réanimation, CHU de Rouen, 76000 Rouen, France; 11 UNIROUEN, Inserm U1096, Normandie Univ, 76000 Rouen, France; 12 Service de réanimation, Centre Cardiologique du Nord, 93200 Saint-Denis, France; 13 Service d’anesthésie réanimation 1, CHU la Timone, Assistance Publique-Hôpitaux de Marseille (AP-HM), 13000 Marseille, France; 14 Service d’anesthésie réanimation sud, centre médico-chirurgical Magellan, CHU de Bordeaux, 33600 Pessac, France; 15 Inserm, UMR 1034, Biology of Cardiovascular Diseases, Univ. Bordeaux, 33000 Bordeaux, France; 16 Service de réanimation polyvalente, Hôpital Erasme, cliniques universitaires de Bruxelles, 1070 Brussels, Belgium; 17 U955-IMRB, Equipe 03 “Pharmacologie et Technologies pour les Maladies Cardiovasculaires (PROTECT)”, Inserm, Univ Paris Est Créteil (UPEC), Ecole Nationale Vétérinaire d’Alfort (EnVA), 94700 Maisons-Alfort, France; 18 Faculté de Santé, Univ Paris Est Créteil, 94010 Créteil, France

**Keywords:** Cardiac surgery, Mediastinitis, Nosocomial infection, Healthcare-associated infection, Fungus, *Candida*, *Aspergillus*, *Trichosporon*

## Abstract

**Objectives:**

The occurrence of mediastinitis after cardiac surgery remains a rare and severe complication associated with poor outcomes. Whereas bacterial mediastinitis have been largely described, little is known about their fungal etiologies. We report incidence, characteristics and outcome of post-cardiac surgery fungal mediastinitis.

**Methods:**

Multicenter retrospective study among 10 intensive care units (ICU) in France and Belgium of proven cases of fungal mediastinitis after cardiac surgery (2009–2019).

**Results:**

Among 73,688 cardiac surgery procedures, 40 patients developed fungal mediastinitis. Five were supported with left ventricular assist device and five with veno-arterial extracorporeal membrane oxygenation before initial surgery. Twelve patients received prior heart transplantation. Interval between initial surgery and mediastinitis was 38 [17–61] days. Only half of the patients showed local signs of infection. Septic shock was uncommon at diagnosis (12.5%). Forty-three fungal strains were identified: *Candida* spp. (34 patients), *Trichosporon* spp. (5 patients) and *Aspergillus* spp. (4 patients). Hospital mortality was 58%. Survivors were younger (59 [43–65] vs. 65 [61–73] yo; *p* = 0.013), had lower body mass index (24 [20–26] vs. 30 [24–32] kg/m^2^; *p* = 0.028) and lower Simplified Acute Physiology Score II score at ICU admission (37 [28–40] vs. 54 [34–61]; *p* = 0.012).

**Conclusion:**

Fungal mediastinitis is a very rare complication after cardiac surgery, associated with a high mortality rate. This entity should be suspected in patients with a smoldering infectious postoperative course, especially those supported with short- or long-term invasive cardiac support devices, or following heart transplantation.

## Introduction

Postoperative mediastinitis is one of the most severe complications after cardiac surgery. The overall incidence of mediastinitis after sternotomy ranges from 0.25 to 5% [1], depending on both surgical procedure and patient’s conditions. Despite advances in cardiac surgery and perioperative care, mortality associated with postoperative mediastinitis remains high, ranging from 8 to 50% depending on the case mix [2, 3]. Although bacteria are the most common cause of mediastinitis [4], fungi are a rising cause of postoperative mediastinitis, with up to 5% of all cases of mediastinitis [5]. However, the true incidence of this disease is difficult to estimate, as most of data come from small cohort studies, and are either recorded as primary infection or coded as superinfection [6]. Compared to bacterial etiologies, fungal mediastinitis appears to be associated with a worse prognosis, leading to frequent systemic dissemination and greater mortality from multiple organ failure [6]. Those findings are in line with studies showing high morbidity and mortality in critically ill patients diagnosed with invasive candidiasis [7]. Other fungi, such as filamentous and emerging yeasts, have also been implicated in postoperative mediastinitis, but the rarity of those forms makes their analysis difficult. So, data on prevalence, presentations and outcome of fungal mediastinitis are clearly lacking after cardiac surgery, making a real gap of knowledge in the fields of perioperative care, cardiac surgery and infectious diseases.

Our multicenter study aimed to describe the characteristics of post-cardiac surgery fungal mediastinitis, to report associated morbidity and mortality and to identify potential factors associated with mortality from these infections.

## Patients and methods

### Study design

Retrospective study in 10 ICU of cardiac surgery centers in France and Belgium.

### Patients

We retrospectively screened patients admitted to ICU during 11 years (01.01.2009 to 30.01.2019).

Each case was extracted through medical charts, using the following keywords: “mediastinitis,” or “sternitis,” or “sternal osteomyelitis,” or “fungal infection,” or “fungemia,” or “deep surgical wound infection,” or “postoperative infection” and “cardiac surgery” and “sternotomy”.

Inclusion criteria were: patient over 18 years of age; patient undergoing cardiac or ascending aorta surgery with sternotomy; occurrence of a post-sternotomy mediastinitis according to the Amsterdam consensus definition and formal identification of a fungus within the surgical site [8]. Post-sternotomy mediastinitis was defined as an infection occurring within one year (regardless of whether an implant is in place or not), and infection appearing related to the operative procedure, with at least one of the following criteria: (1) patient has microorganisms cultured from mediastinal tissue or fluid obtained during a surgical operation or needle aspiration, (2) patient has evidence of mediastinitis confirmed during a surgical operation or histopathological examination, (3) patient has at least one of the following signs or symptoms with no other recognized cause: fever (> 38 °C), chest pain or sternal instability; (4) at least one of the following: purulent discharge from mediastinal area, organisms cultured from blood or discharged from mediastinal area, radiological evidence of an infective process in the mediastinum.

Exclusion criteria were defined as follows: unproven mediastinitis, fungemia from other source, esophageal or cervico-facial surgery requiring sternotomy.

### Variables and outcomes

The following variables were collected: Euroscore II [9], timing of surgery (emergent, urgent, elective), type of surgery (coronary, valvular, ascending aorta surgery, heart transplantation or left ventricular assist device (LVAD) implantation), duration of procedure, duration of cardiopulmonary bypass and aortic cross-clamping (in case of cardiopulmonary bypass), SAPS II (Simplified Acute Physiology Score II) [10] and SOFA (sequential organ failure assessment) scores at initial ICU admission, use of catecholamines, implantation of veno-arterial extracorporeal membrane oxygenation (V-A ECMO), organ failure, mediastinitis and mycological data (type of fungus, species, resistance profile, associated fungemia) and antifungal treatment. Finally, hospital mortality was recorded.

### Statistical analysis

Categorical variables are described as number and percentages, and continuous variables as median [interquartile range]. We compared survivors and non-survivors to identify variables associated with mortality. Comparisons between groups were made using Chi-square test for categorical variables and *t*-test or Mann–Whitney test for continuous variables. *p* value < 0.05 was considered statistically significant. Analyses were performed using R statistical platform, version 3.0.2 (https://cran.r-project.org/).

## Results

### Patient’s characteristics

During this 11-year study period, 40 patients with documented fungal mediastinitis were identified among 73,688 cardiac surgery procedures, that is an incidence of 0.05% of procedures. Main characteristics are depicted in Table 1. Briefly, thirty-two (80%) were men, with a median age of 63 [56–69] years. Main comorbidities were chronic heart failure (72%) and diabetes (50%). Five patients were supported with LVAD and five by peripheral VA-ECMO before initial surgery. The preoperative Euroscore II was 11.8 [4.2–21.2] % and initial ICU admission SOFA score was 9 [5–10]. Eighteen patients underwent coronary artery bypass graft (CABG) or valve replacement surgery, and 12 heart transplantation. Other procedures were LVAD implantation (*n* = 2), total artificial heart (*n* = 2) and thoracic paraganglioma removal requiring cardiopulmonary bypass (*n* = 1).

**Table 1:** Characteristics of patients with post-cardiac surgery mediastinitis

Baseline characteristics	n = 40
Age (yo)	63 [56–69]
Male	32 (80)
BMI (kg/m^2^)	26 [22–32]
Euroscore II (%)	11.8 [4.2–21.2]
SAPS II	44 [30–56]
Admission SOFA	9 [5.5–10.5]
*Comorbidity, n (%)*	
Chronic heart failure	29 (72)
COPD	7 (17)
Chronic kidney disease	6 (15)
Dialysis	1 (2.5)
Diabetes mellitus	20 (50)
Under insulin	10 (25)
Immunosuppression	5 (13)
Including heart transplant recipients	4 (10)
LVAD	5 (13)
Recent hospitalization (< 3 months)	22 (55)
Active smoker	9 (23)
*Timing of initial surgery, n (%)*	
Elective	12 (30)
Emergent	13 (32)
Urgent	15 (37)
*Initial surgery, n (%)*	
CABG	9 (22.5)
Valve replacement	9 (22.5)
Combined (CABG + valve)	5 (12.5)
Heart transplantation	12 (30)
Other	5 (13)
Surgical procedure duration (min)	309 [233–437]
CPB duration (min)	144 [93–206]
ACC duration (min)	88 [61–147]

Data are expressed as number (percentage) or median [IQR], as appropriate

*BMI* Body mass index, *SAPSII* Simplified acute physiology score II, *SOFA* Sequential organ failure assessment, *COPD* chronic obstructive pulmonary disease *LVAD* Left ventricular assist device, *CABG* Coronary artery bypass graft, *CPB* cardiopulmonary bypass, *ACC* Aortic cross-clamping

### Clinical presentation, diagnosis and impact of mediastinitis

Of all the cases, only 20 patients showed clinical local signs suggestive of mediastinitis; the other cases were diagnosed following postoperative fever, raised biological inflammatory markers or positive cultures. Five of the remaining cases had a radiological diagnosis without clinical or biological symptoms. Septic shock was the primary clinical presentation for five patients (Table 2). The median interval between initial surgery and mediastinitis diagnosis was 38 [17–61] days; 22 patients were diagnosed during the initial ICU stay with a median interval of 27 [15–41] days, while patients who were diagnosed after ICU discharge had a median interval of 57 [42–150] days. Eight patients were supported with peripheral VA-ECMO when they developed mediastinitis, and two had LVAD. Nineteen patients developed septic shock after surgical treatment of fungal mediastinitis; seventeen patients required renal replacement therapy.

**Table 2:** Presentation of patients with post-cardiac surgery fungal mediastinitis

Presentation of mediastinitis	*N* (%)
*Clinical signs*	
Local signs (inflammatory/purulent sternotomy)	20 (50)
Fever (temperature > 38°5C)	5 (13)
Septic shock	7 (18)
*Biological signs*	
Biological inflammatory syndrome (WBC > 12 G/L)	7 (18)
Positive mediastinal culture	15 (38)
*Radiological signs*	
Mediastinal collection on CT-scan	7 (18)

*CT-scan* computed tomography scan, *WBC* white blood cell

Data are expressed as number (percentage)

In-hospital mortality was 58% (23 patients). After ICU discharge, hospital length of stay was 31 [21–49] days.

### Microbiological documentation and pathogens

The microbiological documentation was obtained from intraoperative samples in 31 patients, trans-sternal puncture for six patients, superficial sampling for two patients and pericardial puncture for one patient. We identified 43 fungal strains among 40 patients, with three patients having co-infection with two species of *Candida* (Table 3). More than 80% of fungal strains were *Candida* spp., mainly *Candida albicans*. Among the patients infected with *Candida* spp, eight were heart transplant recipients. Other strains were *Trichosporon* spp. (five cases, including two heart transplant recipients) and *Aspergillus* spp. (four cases, including two heart transplant recipients) (Table 3). Six strains were resistant to fluconazole (four *C. glabrata* and two *C. parapsilosis*). Associated fungemia was present in 14 patients (35%).

**Table 3:** Postoperative mediastinitis causative pathogens and time to diagnosis

Microbiological strains (total *n* = 43)	*N* (%)	Mortality (%)	Time to diagnosis (days)
*-Candida* spp.	34 (85)	21 (61)	35 [16–57]
*Candida albicans*	19 (48)	9 (47)	41 [18–68]
Non-*albicans Candida*	15 (3)	11 (80)	34 [15–46]
*Candida parapsilosis*	6 (15)		
*Candida glabrata*	5 (13)		
*Candida tropicalis*	2 (5)		
*Candida lusitaniae*	1 (3)		
*Candida krusei*	1 (3)		
*-Trichosporon* spp.	5 (13)	4 (80)	31 [18–61]
*Trichosporon inkin*	2 (5)		
*Trichosporon ashaii*	1 (3)		
*Trichosporon* undefined	2 (5)		
*-Aspergillus fumigatus*	4 (10)	0 (0)	269 [217–318]

Data are expressed as number (percentage) or median [IQR], as appropriate

To note, delay between initial surgery and the diagnosis of fungal mediastinitis was longer in patients with *Aspergillus* spp*.* mediastinitis, compared to *Candida* spp. mediastinitis (269 [217–318] vs. 35 [16–57] days, respectively, *p* = 0.05) (Table 3).

### Antifungal and surgical treatment

Echinocandins were used as first-line antifungal therapy in 23 (57%) patients, whereas 15 (37%) patients received azoles and three were treated with amphotericin B; one patient received initially a combination of azole and echinocandin (Table 4).

**Table 4:** Antifungal and surgical treatment according to survival

Treatment and outcomes	Survivors (*n* = 17)	Non-survivors (*n* = 23)	*p*	OR
*First-line treatment, n (%)*				
Echinocandin	10 (59)	13 (56)	0.88	
Azole	6 (35)	9 (39)	0.81	
Amphotericin B	1 (6)	2 (9)	0.74	
*Intervention, n (%)*				
Redon vacuum	14 (82)	19 (83)	1	
Open-chest	1 (6)	3 (13)	1	
Mediastinal irrigation	1 (6)	4 (17)	0.62	
Vacuum assisted closure	5 (29)	5 (22)	0.37	
*Organ dysfunction, n (%)*				
Norepinephrine administration more than 48 h	14 (82)	18 (78)	0.26	
Renal replacement therapy	3 (18)	14 (61)	**0.002**	7.2 [1.6–32]
Mechanical ventilation	6 (35)	15 (65)	**0.05**	3.4 [0.9–12.8]
Duration of mechanical ventilation (days)	12 [2–31]	25 [12–40]	0.17	

Significant values are provided in bold

Data are expressed as number (percentage) or median [IQR], as appropriate

Surgical treatment was performed in all but one patient, of which 33 had multiple high-vacuum Redon catheters, four were left open-chest, ten had negative-pressure wound therapy, and five had mediastinal irrigation. More than one surgical debridement was required for 20 patients (50%).

### Trends in incidence of mediastinitis

Comparing the first and second half of the decade of this study (2009–2014 and 2015–2019), we found a threefold increase in the number of fungal mediastinitis cases during the second interval, i.e., 10 and 30 cases, respectively, with corresponding mortality rates of 70% and 53%.

### Survivors versus non-survivors

Considering baseline characteristics, survivors were younger (59 [43–65] vs. 65 [61–73] yo; *p* = 0.013), had lower BMI (24 [20–26] vs. 30 [24–32] kg/m^2^; *p* = 0.028) and lower SAPSII score at admission (37 [28–40] vs. 54 [34–61]; *p* = 0.012). There was no statistically significant difference between the two groups regarding the type or length of surgery, or the surgical treatment used (Table 5).

**Table 5:** Parameters include baseline characteristics, microbiological identification, management and impact of mediastinitis in surviving and dead patients

Characteristics	Survivors (*n* = 17)	Non-survivors (*n* = 23)	*p*	OR
Age (yo)	59 [43–65]	65 [61–73]	**0.013**	1.1 [1.02–1.19]
Male	14 (82)	18 (78)	1	
BMI (kg/m^2^)	24 [20–26]	30 [24–32]	**0.028**	1.21 [1.01–1.47]
Euroscore II (%)	12 [4.2–19.4]	11.7 [4.6–25]	0.84	
SAPS II	37 [28–40]	54 [34–61]	**0.012**	1.07 [1–1.7]
Admission SOFA	9 [5–11]	9 [6–10]	0.93	
*Comorbidity n (%)*				
Chronic Heart failure	12 (70)	17 (74)	1	
COPD	2 (11)	5 (21)	0.68	
Chronic kidney disease	3 (18)	3 (13)	0.79	
Dialysis	1 (6)	0 (0)	0.42	
Diabetes mellitus	8 (47)	12 (52)	0.75	
Insulin	5 (29)	5 (21)	0.57	
Immunosuppression	3 (18)	2 (9)	0.63	
Heart transplanted	2 (11)	2 (9)	1	
Recent hospitalization (< 3 months)	10 (59)	12 (52)	0.68	
Active Smokers	4 (24)	5 (21)	1	
*Initial surgery*				
Type of surgery, *n* (%)				
CABG	3 (18)	6 (27)	0.52	
Valve replacement	4 (24)	5 (22)	1	
Combined (CABG + valve)	1 (6)	4 (17)	0.37	
Heart transplantation	8 (47)	4 (17)	0.08	
LVAD	1 (6)	1 (4)	1	
Other	2 (12)	1 (4)	0.56	
Surgery duration (min)	354 [281–397]	278 [172–457]	0.19	
CBP duration (min)	186 [138–211]	133 [86–185]	0.19	
ACC duration (min)	107[69–166]	87 [51–116]	0.09	

Significant values are provided in bold

Data are expressed as number (percentage) or median [IQR], as appropriate

*BMI* Body mass index, *SAPSII* Simplified acute physiology score II, *SOFA* Sequential organ failure assessment, *COPD* chronic obstructive pulmonary disease, *LVAD* Left ventricular assist device, *CABG* Coronary artery bypass graft, *CPB* cardiopulmonary bypass, *ACC* Aortic cross-clamping

**Fig. 1:**
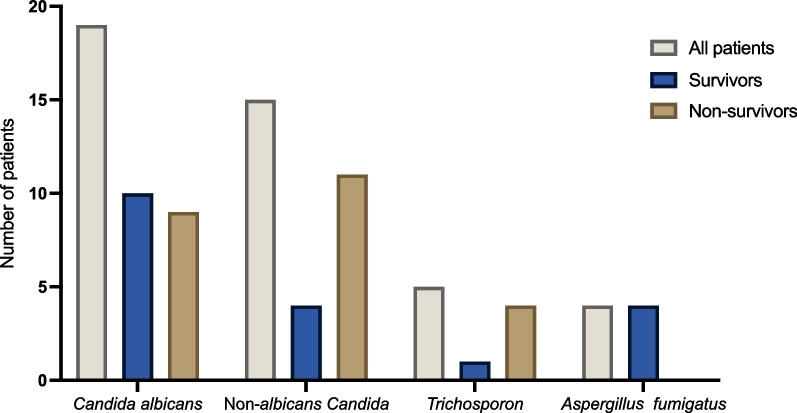
Distribution of fungi among survivors and non-survivors presenting post-cardiac surgery fungal mediastinitis

Non-survivors were more likely to require renal replacement therapy and had prolonged mechanical ventilation (Table 4). Interestingly, time between initial surgery and diagnosis of mediastinitis was longer in survivors (53 [17–166] vs. 34 [17–57] days; *p* = 0.03). Fungal strains among survivors and non-survivors are reported in Fig. 1.

## Discussion

In this 11-year study in high-volume cardiac surgery centers, the incidence of fungal mediastinitis after cardiac surgery was low, accounting for around 0.05% of initial surgical procedures. *Candida* spp. were the main causative agents. Prognosis was poor, with almost two-thirds of patients dying within a month of diagnosis.

Our data bring new insights to the current literature, as this is to date the largest multicenter study on post-cardiac surgery fungal mediastinitis. Indeed, mycological data in critically ill patients are poorly reported and are mainly available for invasive *Candida* and *Aspergillosis* infections, or in hematological population*.*

While some risk factors are shared between fungal and bacterial mediastinitis, such as malnutrition, obesity or diabetes mellitus [11], our study attempted to identify specific factors associated with higher mortality in the fungal variants.

As previously described, a higher Euroscore II and a higher SAPS II score seem to be associated with higher complication rates in patients with fungal mediastinitis. Also, more than half of cases were previously hospitalized before surgery, suggesting a possible association between fungal infection and comorbidities requiring multiple hospital attendance.

With only half of the patients showing local signs at clinical presentation, fungal mediastinitis appears more indolent than bacterial mediastinitis (Table 2). The latter is almost systematically associated with local signs [12]. Finally, time to diagnosis of fungal mediastinitis after surgery looks prolonged compared to bacterial mediastinitis, with a median delay of 38 days [13]. The non-bacterial nature and delayed onset of mediastinitis may explain a lower rate of septic shock in our population, given that some data suggest that early onset of mediastinitis is associated with septic shock [13].

Thirty percent of cases were cardiac transplant recipients with immunosuppressive agents, making them at higher risk of fungal colonization and opportunistic infections. Previous data suggest that the detection of post-cardiac transplant bacterial mediastinitis is very challenging, with lower white blood cell count and fever [14]. Keeping a high level of suspicion for bacterial and non-bacterial healthcare-associated infections is of paramount importance to improve early diagnosis and prognosis. Our data suggest that the diagnosis of fungal mediastinitis may be significantly delayed and that septic shock in this population occurs latter in about 50% of cases. In order to reduce the diagnosis delay, the fungal wall biomarker β-D-glucan could regularly be assayed in high-risk patients, notably V-A ECMO or heart transplants recipients. If this biomarker could be useful for *Aspergillosis* spp. and *Candida* spp., it has never been evaluated in this setting and cannot be used in case of *Trichosporon* spp. infections.

After initial surgery, one in five patients was supported by V-A ECMO for cardiogenic shock when mediastinitis was diagnosed. Although the higher prevalence of fungal infections on V-A ECMO remains controversial [15, 16], circulatory support reflects a more severe patient condition, leading to a potential increased susceptibility to infections.

Regarding ventricular mechanical supports, it is important to highlight that five patients received LVAD before initial surgery, and that all benefited from transplantation. Three of them developed postoperative mediastinitis due to fungal pathogens, mostly non-*Candida* species. LVAD as destination therapies are at risk of infectious complications, through infection of the percutaneous site or pocket infection [17], and prior LVAD before heart transplantation has been identified as a supplementary risk factor of bacterial mediastinitis after cardiac transplantation [1]. These facts may suggest a specific vulnerability to fungal mediastinitis among transplanted patients previously on prolonged heart assistance [18]. Notably, the role of per and postoperative antifungal prophylaxis to prevent infection in patients receiving long-term assist device or heart transplantation is still a matter of debate.

We presume that airborne contamination from *Aspergillus* spp. spores may occur during the surgical procedure [19]. Spreading in the mediastinal area from a contiguous source or an hematogenous invasion is also conceivable, especially among immunosuppressed patients [20]. For *Candida* spp. and *Trichosporon* spp., direct inoculation from skin barrier rupture during surgery or cross-contamination is theoretically possible [21].

The most common strains were *Candida* spp. The subspecies were consistent with the current *Candida* distribution described in critically ill patients [22]. To note, *Aspergillus* mediastinitis is a very rare condition after cardiac surgery, with only few case reports [23–25]. In our study, only four patients had postoperative *Aspergillus* mediastinitis and none of them died in ICU.

We reported five cases of postoperative mediastinitis caused by *Trichosporon* spp.; four out of five patients died from these infections, which makes this opportunistic pathogen the deadliest strain with non-*albicans Candida*, with a reserve of anecdotal evidence. However, the number of cases prevents us from drawing any conclusion about the respective virulence of these pathogens.

There are no specific recommendations to guide the management of fungal mediastinitis. In our study, all but one patient had surgical treatment, which is a cornerstone of the management of postoperative bacterial mediastinitis [26]. Moreover, half of the patients required at least a second surgical debridement, underlining the difficulties in obtaining satisfying source control.

Most of patients were treated with echinocandin or azoles as a first-line antifungal therapy. However, we found that 15% of fungal strains were resistant to azoles, which mainly involved *C. glabrata* and *C. parapsilosis*. Whereas fluconazole resistance is already described for these two species [27, 28], our data strengthen the need to carefully choose the empirical antifungal therapy.

There is increasing concern in the literature, suggesting that non-*albicans Candida* can generate a biofilm, yielding issues to remove the fungal burden [29]. However, it seems that these strains remain sensitive to echinocandin [30]. Additionally, our results suggest the need to consider non-*albicans Candida* species when choosing first-line antifungal treatment. However, most of therapeutic suggestions are adapted from other deep fungal surgical site infections, and proposed from local experiences and based on limited series. No randomized trial could be built to answer to this too rare condition, due to a likely ultra-low recruitment rate. The limited evidence for medical treatment can be extrapolated from recommendations for the management of osteomyelitis and endocarditis candidiasis.

Postoperative *Candida* mediastinitis should be treated by surgical debridement, followed by echinocandin or fluconazole in the absence of invasive circulatory support and in the absence of underlying aorta prosthesis. As recommended for endocarditis, the preferred treatment among patients with invasive devices or vascular prosthesis is either lipid formulation amphotericin B (sometimes associated with flucytosine), or high dose echinocandin. Step-down therapy to fluconazole can be considered in patients who have fluconazole-susceptible *Candida* isolates and quite stable clinical condition, that is after 2 weeks of initial amphotericin or echinocandin treatment. Optimal treatment duration is unknown, but usually requires several months. For ventricular assist devices that cannot be removed, chronic suppressive therapy with fluconazole is recommended, if the isolate is susceptible, as long as the device remains in place [31].

In cases of *Aspergillus* spp. mediastinitis, voriconazole and liposomal amphotericin B should be preferred as first-line antifungal therapy, analogous to the treatment of extrapulmonary aspergillosis. Long-term treatment with oral voriconazole is recommended after initial therapy [32]. Eventually, *Trichosporon* spp*.* mediastinitis medical treatment relies on voriconazole or posaconazole, given the fact that these yeasts are intrinsically resistant to echinocandins [33]. This point is of utmost importance in the critically ill patients, because guidelines recommend favoring empiric echinocandins treatment in patients with invasive fungal infection [31].

In addition, high levels of suspicion for fungal mediastinitis should be kept in patients with perioperative clinical evidence of mediastinitis and negative bacteriological cultures, especially in those who received heart transplantation. In this population, intraoperative surgical samples should be sent for mycological analysis, in addition to the usual bacteriological analysis.

Whereas the usual mortality rate of postoperative mediastinitis ranges from 30 to 50% [3], our study highlights a higher mortality rate of nearly 60%. Fungal infections in the critically ill patients are associated with a high mortality rate, even if this reflects a large spectrum ranging from putative pulmonary aspergillosis to invasive candidiasis in hematological patients. Indeed, in the overall critically ill patients, invasive *Candida* infections are associated with roughly 50% mortality rate [34]. A recent single-center retrospective study focusing on *Candida* spp. postoperative mediastinitis underlined a significantly lower survival than bacterial mediastinitis (43 ± 8% vs. 80 ± 6.3%, respectively; *p* < 0.0001) [35]. One explanation might be the high prevalence of ECMO-supported patients (62%) and the over-representation of cardiac transplantation recipients in this cohort. However, this hypothesis must be interpreted with caution, since specific data on fungal mediastinitis is limited.

In our study, a short delay between surgery and infection was related to mortality. This finding was previously described in patients with post-sternotomy bacterial mediastinitis [13]. The early postoperative decrease and loss of function of lymphocytes [34] leads to an increased vulnerability to infection. Except for *Aspergillus* spp. infections which have a longer time to onset, we did not find any difference in terms of infection delay between the causative agents.

Notably, we found an increased trend of post-cardiac surgery fungal mediastinitis between 2009 and 2019. This observation corroborates a similar tendency in pulmonary fungal infections and in general fungal disease [36]. This could be related to an increased population of immunocompromised patients, including those who receive immunomodulatory agents. The indications of V-A ECMO have also largely increased worldwide over the last decade, exposing this high-risk population to nosocomial infections. It is noteworthy that the lack of record of actual number of VA-ECMO, LVAD or heart transplantation during the study period prevents us from providing trends of surgical procedures and specific complications in our centers. Moreover, the improvement of the diagnosis techniques of fungal infections [37] may have contributed to a greater identification rate of fungal mediastinitis, and clinicians awareness’s may have been raised by previous experiences. To note, no change in national or local antibioprophylaxis policy occurred during the study period. So, the changes in case mix over time are likely the predominant factor explaining the increasing incidence.

Overall, evidence-based recommendations about treatments and survival following this cardiac surgical complication are likely to be uncertain, due to methodology issues and extremely low incidence. Our initiative opens the door to a larger sample experience with recruitment of international centers, in order to better appreciate “real-life” epidemiology, outcome and treatment algorithms.

Our study presents several limitations. First, the inclusion period of these 40 cases lasted 11 years in 10 centers, involving potential changes related to surgical and medical management over the years. However, the extremely low incidence in our cohort would be unsuitable for prospective studies. Identification of mediastinitis may have differed between centers, and cases may have been underdiagnosed or diagnosed in other centers. So, this incidence should be viewed as an estimation. Whatever, it is very likely that the incidence would stay very low. Similarly, due to the sample size, no multivariable regression was possible to identify risk factors of onset. Second, our population was heterogeneous, with cardiac transplantation representing a specific immunocompromised population which should probably be considered aside. Our study would have benefited from comparing patients after each specific procedures, notably, coronary/valvular surgery, ECMO/LVAD/heart transplantation recipients, so as to better identify specific patient or procedure risk factors. Larger database will have to indicate the volume of each surgical procedure. Third, we focused on post-cardiac surgery fungal mediastinitis, which does not allow the generalization of our results to other postoperative mediastinitis, including mediastinitis after esophageal or cervico-facial surgery. Finally, in the absence of a control population of bacterial mediastinitis, we could not draw firm comparison between fungal and non-fungal mediastinitis: assertions regarding clinical presentations, risk factors and outcomes should be cautiously considered.

## Conclusion

Fungal mediastinitis is an extremely rare but serious complication after cardiac surgery, with an ICU mortality rate of more than 50%. While *Candida* spp. are the leading pathogens, our results are in line with a trend toward an increased prevalence of emergent yeasts and molds that cause infectious complications.

We suggest holding a high level of suspicion among patients presenting with sepsis after cardiac surgery, especially in case of latent, scarcely inflammatory sternal aspect and underlying immunodepression. An empiric antifungal therapy should be discussed in heart transplant recipients who have evidence of mediastinitis but early negative bacteriological culture.

Further studies including larger samples and comparison groups are mandatory to promote more evidence-based strategies. An algorithm implemented by all involved specialists is likely to guide clinicians and strengthen analysis through standardization.

## Data Availability

The storage of anonymized data is computerized and centralized at the principal investigator's office, which guarantees their protection. Data could be shared on reasonable request to the corresponding author.

## Abbreviations

BMI: Body mass index
CABG: Coronary artery bypass graft
ICU: Intensive care unit
LVAD: Left ventricular assist device
SAPS II: Simplified Acute Physiology Score II
SOFA: Sequential organ failure assessment
V-A ECMO: Veno-arterial extracorporeal membrane oxygenation
